# Turning waste gases into valuables

**DOI:** 10.1016/j.synbio.2022.04.002

**Published:** 2022-04-04

**Authors:** Huawei Zhu, Yin Li

**Affiliations:** CAS Key Laboratory of Microbial Physiological and Metabolic Engineering, State Key Laboratory of Microbial Resources, Institute of Microbiology, Chinese Academy of Sciences, Beijing, 100101, China

Waste off-gases from steel mills and other mining related industries often contain rich carbon monoxide (CO), carbon dioxide (CO_2_), and sometimes hydrogen (H_2_). The biological conversion of waste gases into chemicals is an absolutely carbon-negative process. To date, the only chemical that can be commercially produced from fermentation of waste gas is ethanol, an important two-carbon (C2) chemical generally used as a blending component of gasoline [1]. This technology was developed by LanzaTech Inc. and has been operating on a commercial scale since 2018. Recently, another breakthrough in gas fermentation was reported by Liew et al. [2] from the same company. They achieved pilot-scale production of acetone and isopropanol from steel mill off-gas by using an engineered acetogen *Clostridium autoethanogenum*.

Different from ethanol, acetone and isopropanol are non-native products of *C. autoethanogenum*. To engineer *C. autoethanogenum* for efficient production of acetone and isopropanol, the authors developed a series of high-throughput strain engineering strategies. In the pathway level, a large combinatorial plasmid library composed of 30 heterologous genes and three promoters were modularly constructed with the assistance of a pMTL80000 vector system, which expedites the process of pathway enzyme screening. In the strain level, by applying a self-developed iPROBE (in vitro Prototyping and Rapid Optimization of Biosynthetic Enzymes) approach [3], the authors identified three effector genes responsible for byproducts accumulation from 13 candidate genes in few days, which bypassed the laborious work of deleting all candidate genes. The final engineered strain consists of about 10 genomic modifications, which is a remarkable achievement for *Clostridium*. These valid genetic modifications ensured the production of C3 chemicals with high productivity (2.5 g/L/h and 3.0 g/L/h for acetone and isopropanol, respectively). The range of titers reached up to 9.2–60.0 g/L for acetone and 11.1–72.0 g/L for isopropanol, which were calculated from the medium dilution rates given in the text [2]. The selectivity of both products reached 80–90%, which were comparable to the native products acetate and ethanol produced in acetogens.

The gas fermentation relies on Wood-Ljungdahl pathway where two one-carbon units (CO or CO_2_) are condensed to form one two-carbon molecule of acetyl-CoA [4]. With acetyl-CoA as a building block, a variety of compounds with variable chain length and reduction degree can be synthesized in a microbial chassis. For production of C2 chemicals like ethanol, the theoretical molar carbon efficiency from C1 gases is 100%. However, in the case of producing C3 chemicals like acetone and isopropanol, the molar carbon efficiency is only 75% because 1 mol of CO_2_ will be released when 1 mol acetone or isopropanol is generated. Thus, once the molar ratio of CO_2_:CO that are fixed into the acetyl-CoA molecule is lower than 1:3, the relative proportion of CO_2_ in the vent gas will be higher than that in the feed gas, meaning net CO_2_ emission occurs. In such a circumstance, the change of the relative proportion of CO_2_ in the feed gas and vent gas should be considered when calculating the GHG emission of gas fermentation.

The energetics of fixing CO and CO_2_ are different since CO is an energy-containing molecule while CO_2_ needs additional energy input [5]. In gas fermentation, the energy is generally supplied by hydrogen (H_2_) present in many feed gases or can be supplied by external H_2_ sources. Theoretically, the supply of H_2_ to the fermentation process would increase the carbon fixation efficiency from CO. When there is sufficient H_2_ serving as the energy source, CO_2_ can be used as the sole carbon source (Table 1). To achieve the maximum conversion of C1 gases into carbon-derived products, efficient H_2_ utilization is crucial. For a given acetogen, elucidating the metabolic characteristics in different gas mixture would also be very useful. To this end, one can track the reaction with ^13^C-labeled CO or CO_2_ under different partial pressure of H_2_. Meanwhile, reinforcing hydrogen oxidation by overexpressing H_2_-uptake hydrogenases is an important strategy for engineering the next-generation gas-fermenting strains [6].

**Table 1:** The carbon efficiency for acetone production with different H_2_:CO ratio scenario.

Composition	Stoichiometry	Δ_r_G^o^ (KJ/mol)	H_2_:CO ratio	Carbon efficiency
**CO**	8 CO + 3 H_2_O → C_3_H_6_O + 5 CO_2_	−320.1	0:8	37.5%
**CO, H**_**2**_	1 H_2_ + 7 CO + 2 H_2_O → C_3_H_6_O + 4 CO_2_	−301.1	1:7	42.8%
**CO, H**_**2**_	2 H_2_ + 6 CO + 1 H_2_O → C_3_H_6_O + 3 CO_2_	−282.0	2:6	50%
**CO, H**_**2**_	3 H_2_ + 5 CO → C_3_H_6_O + 2 CO_2_	−263.0	3:5	60%
**CO, H**_**2**_	4 H_2_ + 4 CO → C_3_H_6_O + H_2_O + CO_2_	−243.9	4:4	75%
**CO, H**_**2**_	5 H_2_ + 3 CO → C_3_H_6_O + 2 H_2_O	−224.9	5:3	100%
**CO, CO**_**2**_**, H**_**2**_	6 H_2_ + 2 CO + 1 CO_2_ → C_3_H_6_O + 3 H_2_O	−205.8	6:2	100%
**CO, CO**_**2**_**, H**_**2**_	7 H_2_ + 1 CO + 2 CO_2_ → C_3_H_6_O + 4 H_2_O	−186.8	7:1	100%
**CO**_**2**_**, H**_**2**_	8 H_2_ + 3 CO_2_ → C_3_H_6_O + 5 H_2_O	−167.7	8:0	100%

Although H_2_ is an inherent energy source in most of waste gases, it was demonstrated to be a thermodynamically unfavorable electron source when compared with CO (Table 1) [7]. Another shortcoming is its low solubility, making high local concentration difficult to achieve unless the bioreactor is pressurized. Thus, exploring other forms of energy sources, such as H_2_S, cathodic current, even light, holds great promise for efficient biomanufacturing of chemicals from waste gases [8].

## References

[1] Heffernan J.K., Valgepea K., Lemgruber R.D.P., Casini I., Plan M., Tappel R., Simpson S.D., Kopke M., Nielsen L.K., Marcellin E. (2020). Enhancing CO2-valorization using Clostridium autoethanogenum for sustainable fuel and chemicals production. Front Bioeng Biotechnol.

[2] Liew F.E., Nogle R., Abdalla T., Rasor B.J., Canter C., Jensen R.O., Wang L., Strutz J., Chirania P., De Tissera S., Mueller A.P., Ruan Z.H., Gao A.L., Tran L., Engle N.L., Bromley J.C., Daniell J., Conrado R., Tschaplinski T.J., Giannone R.J., Hettich R.L., Karim A.S., Simpson S.D., Brown S.D., Leang C., Jewett M.C., Kopke M. (2022). Carbon-negative production of acetone and isopropanol by gas fermentation at industrial pilot scale. Nat Biotechnol.

[3] Karim A.S., Dudley Q.M., Juminaga A., Yuan Y.B., Crowe S.A., Heggestad J.T., Garg S., Abdalla T., Grubbe W.S., Rasor B.J., Coar D.N., Torculas M., Krein M., Liew F., Quattlebaum A., Jensen R.O., Stuart J.A., Simpson S.D., Kopke M., Jewett M.C. (2020). In vitro prototyping and rapid optimization of biosynthetic enzymes for cell design. Nat Chem Biol.

[4] Yang C.L., Dong L.F., Gao Y.H., Jia P., Diao Q.Y. (2021). Engineering acetogens for biofuel production: from cellular biology to process improvement. Renew Sustain Energy Rev.

[5] Bertsch J., Muller V. (2015). Bioenergetic constraints for conversion of syngas to biofuels in acetogenic bacteria. Biotechnol Biofuels.

[6] Claassens N.J., Sousa D.Z., dos Santos V., de Vos W.M., van der Oost J. (2016). Harnessing the power of microbial autotrophy. Nat Rev Microbiol.

[7] Hu P., Bowen S.H., Lewis R.S. (2011). A thermodynamic analysis of electron production during syngas fermentation. Bioresour Technol.

[8] Clomburg J.M., Crumbley A.M., Gonzalez R. (2017). Industrial biomanufacturing: the future of chemical production. Science.

